# Public Health in the Time of Bioterrorism

**DOI:** 10.3201/eid0810.020444

**Published:** 2002-10

**Authors:** Bradley A. Perkins, Tanja Popovic, Kevin Yeskey

**Affiliations:** * Centers for Disease Control and Prevention, Atlanta, Georgia, USA

On Thursday, October 4, 2001, just 24 days after the tragic events of September 11, the Florida Department of Health and the Centers for Disease Control and Prevention (CDC) confirmed the first case of inhalational anthrax in the United States in more than 25 years. Recognition of this unexpected case is attributed to the alertness of local infectious disease physician Larry Bush, who promptly notified Jean Malecki, director, Palm Beach County Health Department (1,2). By Saturday, October 6, a team of federal, state, and local public health and local law enforcement investigators identified intentional *Bacillus anthracis* spore contamination at the patient’s workplace. These events marked the beginning of the first U.S. outbreak of bioterrorism-related anthrax and (for many of us in clinical medicine, public health, and law enforcement) ushered in the transition from tabletop bioterrorism exercises to real-world investigation and response.

Contingency plans to mitigate bioterrorism-related anthrax outbreaks go back to August 1998, when CDC hosted the “Workshop on Improving Public Health Response to Possible Acts of Bioterrorism.” This workshop brought together state and local health departments, public health professional organizations, the U.S. Department of Defense, and the U.S. Department of Justice to examine ways of improving public health preparedness for bioterrorism (CDC, unpub. data). Two investments made as a result of this workshop were the Laboratory Response Network for Bioterrorism and the National Pharmaceutical Stockpile. These early investments were key components of the public health response to the 2001 bioterrorism-related anthrax outbreak.

The Laboratory Response Network was created at the recommendation of the 1998 Workshop’s “Diagnosis Working Group,” the then Association of State and Territorial Public Health Laboratories (now Association for Public Health Laboratories), and CDC. The Laboratory Response Network is a tiered system of laboratories with capacities defined in an A (lowest tier) through D (highest) pyramid structure (3,4). In support of this structure, procedures for identification of *B. anthracis*, and other Category A biologic agents, were validated, and in some instances developed (or redeveloped) de novo on the basis of older methods. Protocols were written into standard laboratory procedure manuals. Reagents for testing were standardized, produced, and distributed by CDC to participating laboratories. State health department laboratory scientists were trained to use these methods for identifying *B. anthracis*, *Yersinia pestis* (causative agent of plague), and *Francisella* *tularensis* (causative agent of tularemia) in the fall and winter of 2000. Capacity for specialized or more developmental diagnostic and other tests for *B. anthracis* (e.g., real time polymerase chain reaction [PCR] [5], direct fluorescent-antibody assay [6], immunohistochemical testing, molecular subtyping [7], and antimicrobial susceptibility testing [8]) were established at CDC and (in some instances) at a small number of other advanced U.S. laboratories (e.g., U.S. Army Medical Research Institute of Infectious Diseases, Fort Detrick, Frederick, Maryland; Department of Biological Sciences, Northern Arizona University, Flagstaff, Arizona). For serologic testing, which was found to be invaluable in identifying anthrax cases during the investigation, existing tests developed for vaccine evaluation were adapted for diagnostic purposes (9). All these laboratory measures were in place before the 2001 anthrax outbreak.

During the acute phase of the outbreak, Laboratory Response Network laboratories processed >121,700 specimens for *B. anthracis* (the bulk from environmental specimens from areas of suspected or confirmed contamination). Public Health Laboratories (other than CDC) tested 84,000 (69%) specimens; the Department of Defense tested 30,200 (25%) specimens; and CDC tested 7,500 (6%) (CDC, unpub. data). Handling the unusual surge of demand without the support of the Laboratory Response Network is difficult to imagine and would have likely compromised the investigation.

The National Pharmaceutical Stockpile was another investment made as a result of the 1998 Workshop and put in place before the 2001 outbreak. During the outbreak, the pharmaceutical stockpile team transported not only antibiotics, anthrax vaccine, clinical and environmental samples, and *B*. *anthracis* isolates but also epidemiologists, laboratory scientists, pathologists, and specialized teams of researchers. Under extreme pressure, the team made 143 sorties to 9 states and delivered 3.75 million antibiotic tablets from October 8, 2001 to January 11, 2002 (CDC, NPS Program Logistics Log, Oct 2001–Jan 2002).

Other earlier public health investments that paid off during the anthrax outbreak investigation were CDC’s more than 50-year-old applied epidemiology training program, Epidemic Intelligence Service, and other academic, state and local health department, and CDC efforts to develop the seasoned cadre of field epidemiologists (10,11) that make up the core of public health investigation and response. These epidemiologists, who work in established networks and make up and often lead complex partnerships, comprise the public health front lines of the bioterrorism response team.

The complexity of the 2001 anthrax investigation and response challenged even experienced field epidemiologists. At the state and federal levels, “incident command”-style management structures were used to address the constant emergence of new information, pursue many public health activities simultaneously across multiple investigations, and communicate effectively. These management structures, which have been adopted by the disaster management and law enforcement communities, are less familiar to public health workers. With some variation from site to site, a typical field investigation structure included local, state, and federal public health partners working on the following teams: Epidemiologic Investigation (what happened?), Intervention (post-exposure prophylaxis and follow-up), Surveillance (identify additional cases), Clinical Evaluation (rapidly evaluate suspect cases), Environmental Assessment (environmental sampling and processing), Remediation (working with the Environmental Protection Agency), and Communication (with the public, partners, and press). These teams were sometimes complemented with Federal Bureau of Investigation (FBI) liaisons; in some cases, public health officials were assigned to FBI investigation teams (12). A senior epidemiologist was also posted to FBI Headquarters in Washington, D.C.

After the October 12 recognition of cutaneous anthrax in New York (13), an emergency operations center was established at CDC, Atlanta, Georgia, to coordinate the outbreak investigation and response. The center tasked more than 2,000 employees (in the field or at headquarters in Atlanta) (CDC unpub. data) to specific functions, including 24**-**hour response capacity with telephone information and call-triage services and other specialized teams (14). CDC/Atlanta-based teams led by senior epidemiologists supported each field investigation team in involved jurisdictions (Florida, New York, Washington D.C., New Jersey, and Connecticut). These teams were in direct and frequent communication with their respective field team about laboratory results, other investigations, and policy decisions. Other teams included the following: Clinical Medicine (evaluation of suspected cases, post-exposure prophylaxis and treatment recommendations) (15–21); Environmental Assessment (evaluation of suspected or confirmed areas of environmental contamination); International Support (22,23); Laboratory Support (coordination across CDC laboratories and the Laboratory Response Network); National Pharmaceutical Stockpile (antibiotics, vaccine, specimens, and people transport); Postal Service Liaison (partnership with the U.S. Postal Service—CDC also assigned a senior epidemiologist to the Postal Service); and State Liaison (to coordinate requests from states without confirmed anthrax cases) (24). Beginning on October 12, CDC’s Morbidity and Mortality Weekly Report published a series of reports, notices, and guidelines as events unfolded (25).

Many unknowns confronted the public-health response team during the anthrax investigation (26). The basics about exposure to *B. anthracis–*contaminated envelopes specifically sent to media outlets and government leaders were understood quickly, given the events in Florida, New York, and then Washington, D.C. (13). Difficulties arose in characterizing anthrax risk to individuals and groups with suspected or confirmed exposure to *B. anthracis–*contaminated envelopes or environments (27). Challenges also arose in the evaluation of *B. anthracis-*containing powders, epidemiologic investigation (28), environmental assessment (29,30) and remediation, surveillance (31,32), diagnosis, treatment, and post-exposure prophylaxis (33–35).

Work with *B. anthracis–*contaminated goat hair in textile mills more than 40 years ago provided some data about the risk of *B. anthracis* spore-containing particles in naturally contaminated occupational environments. These data suggested that relatively high levels of *B. anthracis* spores were “not necessarily or consistently dangerous” in this setting (36). Biologic warfare experts considered it unlikely that terrorists could produce a *B. anthracis* spore powder for use in an envelope that would be capable of generating substantial primary (or secondary) aerosol threats for human infection or widespread contamination of environments. Yet, in Senator Daschle’s office, in the Hart Senate Office building, in the room where the letter was opened (as well as outside the room) exposed persons’ nasal mucosa were almost immediately contaminated (37). Re-aerosolization (secondary aerosol) at a level consistent with potential transmission was demonstrated off the implicated high-speed sorter in the Brentwood Processing and Distribution Facility (38). Recent research using simulates of *B. anthracis* spores from the Canadian Defense Establishment Suffield suggests that contaminated envelopes can cause heavy aerosol contamination (39). New understanding is accumulating, and this should improve public health response in the future.

The decision-making involved in closing the U.S. Postal Service’s Brentwood Processing and Distribution Facility, Washington, D.C., has been criticized. The risk to Brentwood facility employees by contaminated envelopes in transit was not recognized in time to prevent illness in four employees, two of whom died (40). Decisions concerning the Brentwood facility were based on epidemiologic observations in Florida and New York, where no disease occurred among postal workers. A possible explanation for the differential risk is that the *B. anthracis* spore preparation in the October 9 envelopes had a higher potential for aerosolization than the preparation in the September 18 envelopes or that the two mailings were made under or exposed to different environmental conditions (e.g., amount of moisture) that created a different potential for aerosolization. A different aerosolization potential is supported by the epidemic curve in the manuscript by Jernigan et al. (13), which shows a higher proportion of inhalational (versus cutaneous) anthrax cases associated with the October 9 mailing. In naturally occurring disease, once risk is understood, it generally remains constant; however, in intentional contamination, risk may be altered by the perpetrator(s).

During the anthrax investigation, the public health response team was better prepared in some areas than in others. Five deaths were not prevented, but widespread illness and death was averted through early recognition of threats and prompt intervention. We applied what we knew and learned what we did not know. We gained new appreciation for communication and partnerships. For the first time, on November 8, 2001, a sitting President of the United States of America, George W. Bush, visited CDC to support the efforts of public health professionals and others who participated in the anthrax investigation and response. Leaders and individual heroes rose in the ranks of public health, clinical medicine, and law enforcement (41). The substantial role of public health in the 2001 anthrax investigation and response suggests that strong public health infrastructure supported by applied public health and basic-science research are key elements to the control and prevention of future bioterrorism threats.

**Figure 1:**
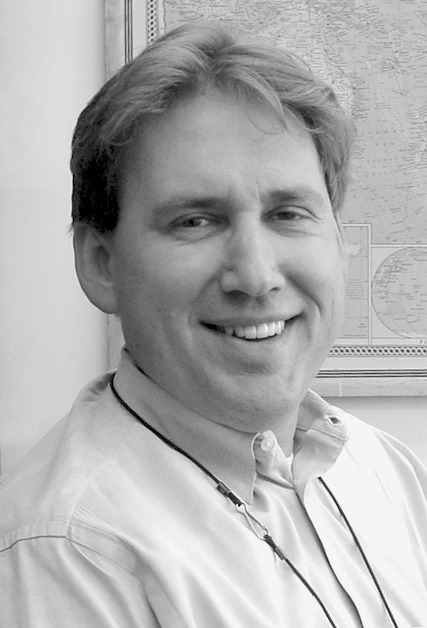
**Bradley A. Perkins, Guest Editor**. Dr. Perkins is chief, Meningitis and Special Pathogens Branch, Division of Bacterial and Mycotic Diseases, Centers for Disease Control and Prevention (CDC), which has technical responsibility for the epidemiologic and laboratory aspects of *Bacillus anthracis*, and selected other bacterial agents of public health importance. Dr. Perkins led the CDC field team in the investigation of the index case of inhalational anthrax in Florida and participated broadly in the 2001 anthrax investigation and response. His research interests include vaccine evaluation, bacterial meningitis, bioterrorism, and emerging infectious diseases. He has worked extensively on the control and prevention of meningococcal disease in the United States, Africa, and around the globe

**Figure 2:**
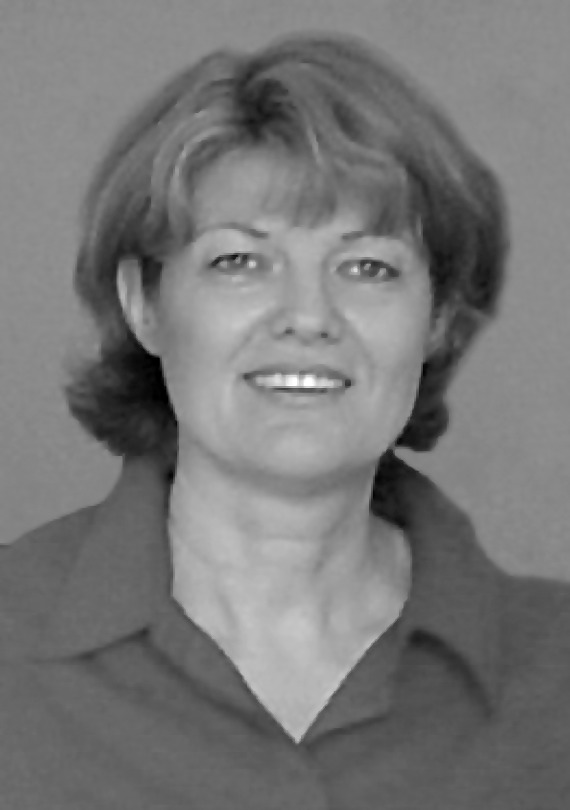
**Tanja Popovic, Guest Editor.** Dr. Popovic is chief, Epidemiologic Investigations Laboratory, Meningitis and Special Pathogens Branch, Division of Bacterial and Mycotic Diseases, Centers for Disease Control and Prevention. As the subject matter expert on laboratory aspects of *B. anthracis* and anthrax at CDC, she and her staff trained laboratory scientists in all 50 states to isolate and identify *B. anthracis* using standard methodologies in the fall of 2000, and have performed thousands of tests for isolation of *B. anthracis*, its confirmatory identification and molecular subtyping during the 2001 anthrax investigation. In addition to bioterrorism preparedness and response, her research focuses on laboratory diagnosis and molecular epidemiology of bacterial meningitis and diphtheria.

**Figure 3:**
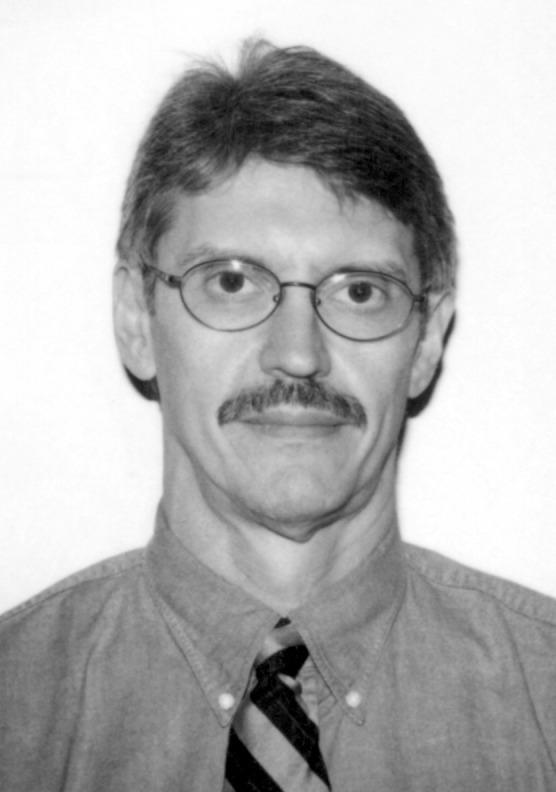
**Kevin Yeskey, Guest Editor.** Dr. Yeskey is director, Bioterrorism Preparedness and Response Program, National Center for Infectious Diseases, Centers for Disease Control and Prevention (CDC). He has served as deputy director of Emergency Public Health in the Division of Emergency and Environmental Health Services, National Center for Environmental Health, CDC. His previous assignments include associate professor and vice chair, Department of Military and Emergency Medicine, Uniformed Services University School of Medicine, and chief medical officer, United States Public Health Service Office of Emergency Preparedness. Dr. Yeskey’s experience with disaster response includes work on hurricanes, earthquakes, floods, mass migrations, and terrorist bombings.
